# A Greek Validation Study of the Multiple Sclerosis Work Difficulties Questionnaire-23

**DOI:** 10.3390/healthcare9070897

**Published:** 2021-07-15

**Authors:** Christos Bakirtzis, Artemios Artemiadis, Elli Nteli, Marina Kleopatra Boziki, Maria-Valeria Karakasi, Cynthia Honan, Lambros Messinis, Grigorios Nasios, Efthimios Dardiotis, Nikolaos Grigoriadis

**Affiliations:** 1 Multiple Sclerosis Center, Second Department of Neurology, Aristotle University of Thessaloniki, GR 54124 Thessaloniki, Greece; nteli.elli@gmail.com (Ε.Ν.); bozikim@auth.gr (M.K.B.); ngrigoriadis@auth.gr (N.G.); 2 Faculty of Medicine, University of Cyprus, Nicosia CY 2029, Cyprus; artemiadis.artemios@ucy.ac.cy; 3 Third University Department of Psychiatry, AHEPA University General Hospital, GR 54124 Thessaloniki, Greece; valeria28289@hotmail.gr; 4 School of Psychological Sciences, College of Health and Medicine, University of Tasmania, TAS 7250 Launceston, Australia; cynthia.honan@utas.edu.au; 5 Neuropsychology Section, University Hospital of Patras, GR 26504 Patras, Greece; lmessinis@upatras.gr; 6 Department of Speech and Language Therapy, University of Ioannina, GR 45110 Ioannina, Greece; nasios@uoi.gr; 7 Department of Neurology, University of Thessaly, GR 41500 Larisa, Greece; edar@med.uth.gr

**Keywords:** multiple sclerosis, employment, patient-reported outcome, MSWDQ-23, validation

## Abstract

The Multiple Sclerosis Work Difficulties Questionnaire-23 (MSWDQ-23) is a self-report instrument developed to assess barriers faced by People with Multiple Sclerosis (PwMS) in the workplace. The aim of this study was to explore the psychometric properties of the Greek version of the MSWDQ-23. The study sample consisted of 196 PwMS, all currently working in part- or full-time jobs. Participants underwent clinical examination and cognitive screening with the Brief International Cognitive Assessment for Multiple Sclerosis (BICAMS) and completed self-report measures of fatigue, psychological functioning, and quality of life, along with the MSWDQ-23 questionnaire. Confirmatory Factor Analysis (CFA) was performed, and goodness-of-fit measures were used to evaluate construct validity. Convergent validity was checked by correlating MSWDQ-23 scores with study measures. Cronbach’s alpha value was produced to assess internal consistency. CFA yielded a model with a fair fit confirming the three-factor structure of the instrument. Higher work difficulties were associated with higher Expanded Disability Status Scale (EDSS) scores, poorer cognitive function, more fatigue, stress, anxiety, and depression, and poorer health status, supporting the convergent validity of MSWDQ-23. Internal consistency (Cronbach’s alpha = 0.94) and test–retest reliability (ICC = 0.996, 95%, CI = 0.990–0.998) were excellent. The Greek MSWDQ-23 can be considered a valid patient-reported outcome measure and can be used in interventions aiming to improve the vocational status of PwMS.

## 1. Introduction

Multiple Sclerosis (MS) is the most common demyelinating and neurodegenerative disease of the central nervous system in young adults [1]. Loss of productivity is common in People with MS (PwMS) and is strongly related to higher levels of physical disability [2,3], reduced subcortical and cortical gray matter volumes [4], cognitive impairment [5], and higher self-perceived fatigue, anxiety, and mood [6,7]. According to an international survey conducted in Europe with more than 13,000 participants, an estimated 50% of working-age PwMS are unemployed, although significant variability of employment rates is observed across countries [8]. According to the results of this survey, cognitive deficits and fatigue substantially impact productivity, even in PwMS with low levels of physical disability. In Greece, the prevalence of MS is currently estimated to be 188.9 per 100,000 inhabitants, totaling approximately 21,000 PwMS [9]. Data about employment rates of PwMS in Greece are however scarce. To our knowledge, only one study has investigated employment in PwMS in Greece. This study found that only 32% out of a sample of 200 PwMS were fully or partially employed [10], indicating that there may be a substantially higher rate of unemployment among PwMS in Greece.

Several international studies have demonstrated the positive effects of engaging in and maintaining employment in PwMS. These positive effects are grounded in the associated improvements with quality of life [11] and self-esteem [12], intellectual enrichment (which enhances cognitive reserve) [13], and avoidance of inactive lifestyles [14], seen in those who are employed. On the contrary, unemployment may lead to social isolation [15], financial dependence on caregivers and social support systems, and relatedly, an inability to cope with disease-related costs [8]. The comprehensive assessment of MS-related work difficulties may therefore enable the identification of the factors that affect the vocational status of PwMS and the likely risk of work withdrawal, as well as the subsequent development of customized vocational rehabilitation programs [16].

The Multiple Sclerosis Work Difficulties Questionnaire 23 (MSWDQ-23) [17] is a shortened version of a 50-item questionnaire that evaluates various domains of working difficulties, categorized into three key dimensions (physical, psychological/cognitive, and external barriers). This 23-item questionnaire has adequate psychometric properties, including good internal consistency and construct validity, and has been validated for use in several languages [18,19,20,21]. This questionnaire enables the detection of MS-related occupational difficulties and has been suggested to be predictive of poor employment outcomes [22,23]. The aim of this study was to validate and assess the psychometric properties of the Greek version of the MSWDQ-23. Common, well-established clinical and cognitive tools, as well as self-report questionnaires were used in this study to assist with this validation. The results of this study indicate that the Greek version of the MSWDQ-23 has sound psychometric properties and maintains the three-factor structure of the original English version.

## 2. Materials and Methods

### 2.1. Study Population

This noninterventional, cross-sectional study was conducted at the Multiple Sclerosis Center of the Aristotle University of Thessaloniki. The study sample consisted of 196 PwMS in full- or part-time employment, recruited from the outpatient clinic. Inclusion criteria were: (a) age ≥ 18 years; (b) MS diagnosis according to the 2017 revised McDonald criteria [24]; (c) being currently employed; (d) the ability to perform all tests and procedures; and (e) no history of any inflammatory event at least 3 months prior to participation. The study was performed according to the Declaration of Helsinki and was approved by the local ethics committee (4.291/4). All participants provided written informed consent prior to their participation.

### 2.2. Tests and Measures

All participants completed a clinical examination by a neurologist where Expanded Disability Status Scale (EDSS) scores were determined, as well as cognitive screening by an experienced neuropsychologist using the Greek version of the Brief International Cognitive Assessment for MS (BICAMS) battery [25], which includes three tests (Symbol Digit Modalities Test (SDMT) [26]; Greek Verbal Learning Test (GVLT) [27]; Brief Visuospatial Memory Test-Revised (BVMT-R) [28]). The BICAMS battery was administered according to the proposed guidelines [29]. Participants also completed the Modified Fatigue Impact Scale (MFIS) to assess self-reported fatigue [30], the Depression, Anxiety, Stress Scale-21 (DASS-21) [31] to assess mood, the Multiple Sclerosis Impact Scale-29 (MSIS-29) [32] to quantify the impact of MS on daily living, and the EuroQoL-5 Dimensions scale (EQ-5D) [33] to assess health-related quality of life. Finally, participants also completed the MSWDQ-23. Prior to use, permission to validate the MSWDQ-23 was obtained by the authors. The COSMIN Study Design checklist for patient-reported outcome instruments (version July 2019) [34] was followed in order to validate this instrument. The questionnaire was translated back and forth by two independent English and Greek bilingual researchers, with all translation discrepancies reviewed on a case-by-case basis until consensus was reached. A final draft version of the questionnaire was trialed in 12 patients (who also provided feedback on the instrument’s readability and comprehension), after which final amendments were made and approved by the study team. Demographic and clinical characteristics and occupational data were also collected. All tests were administered in the same order to all participants, in a quiet room with no distractions. All participants completed the questionnaires without assistance. Finally, 25 randomly chosen participants were re-administered the MSWDQ-23 two weeks later to assess the test–retest reliability of the scale.

### 2.3. Statistical Analysis

Data were checked for deviations from normality by visual inspection of histograms and Q-Q plots. Confirmatory Factor Analysis (CFA) was performed to evaluate the construct validity of the MSWDQ-23 using SPSS v22.0 and AMOS software for Windows (Armonk, NY: IBM Corp). Goodness-of-fit measures (and the corresponding cut-offs showing a good fit) to assess the model fit were the normed χ^2^ (χ^2^/*df*) to overcome the effect of the sample size (cut-off ≤ 3), the Root-Mean-Squared Error of Approximation (RMSEA, cut-off ≤ 0.08, for the 90% CI lower bound ≤ 0.05 and for the 90% CI upper bound < 0.10), the Standardized Root-Mean-squared Residual (SRMR, cut-off ≤ 0.08), and the Comparative Fit Index (CFI, cut-off ≥ 0.9) [35,36]. Items with standardized regression weights or loadings of less than 0.3 were assessed with a view toward removing such items from the model if present. Modification indices for error covariances of conceptually linked items over the value of 10 were identified and accounted for in the final model. In the context of construct validity, we assessed the relationship between the MSWDQ-23 and age (Pearson’s rho correlation) and sex, disease type, education, and work type (the Mann–Whitney U-test was performed for these categorical variables). The absence of floor or ceiling effects was determined by the percentage of scores with maximum or minimum values respectively being less than 15%. Convergent validity was examined by correlating MSWDQ-23 scores with disease status variables (disease duration and EDSS), cognitive test scores (i.e., SDMT, GVLT, BVMT-R), and questionnaire scores (MFIS, DASS-21, MSIS-29, and EQ-5D). Correlation coefficients of >0.7, 0.51–0.7, 0.31–0.5, and 0.1–0.3 were considered very large, large, moderate, and small, respectively [21,37]. Cronbach’s alpha was produced to assess internal consistency. Cronbach’s alpha was also calculated after omitting items one-by-one in each subscale to assess the influence of each item on the subscale’s internal consistency. The Intraclass Correlation Coefficient (ICC) with 95% Confidence Intervals (95% CI) for two-way random-effects models was used to assess test–retest reliability. A significance level of <0.05 was used for all analyses.

## 3. Results

### 3.1. Sample Characteristics

The study sample consisted of 126/196 (64.3%) females. The mean age was 38.6 years (SD = 10.0, range = 19–66). In total, 173/196 (88.3%) had Relapsing–Remitting MS (RRMS), 14/196 (7.1%) had Secondary Progressive MS (SPMS), and 9/196 (4.6%) had Primary Progressive MS (PPMS). There were 47/196 (24%) participants who completed primary/secondary education, while the remainder (76%) were educated at the tertiary level. The majority of the participants (107/196, 54.6%) were in full-time employment (i.e., working 8 or more hours per day) with the remainder in part-time employment (89/196, 45.4%). There were no missing values regarding the MSWDQ-23 instrument. Demographic and disease characteristics of the participants are presented in Table 1.

### 3.2. Confirmatory Factor Analysis

**Figure 1:**
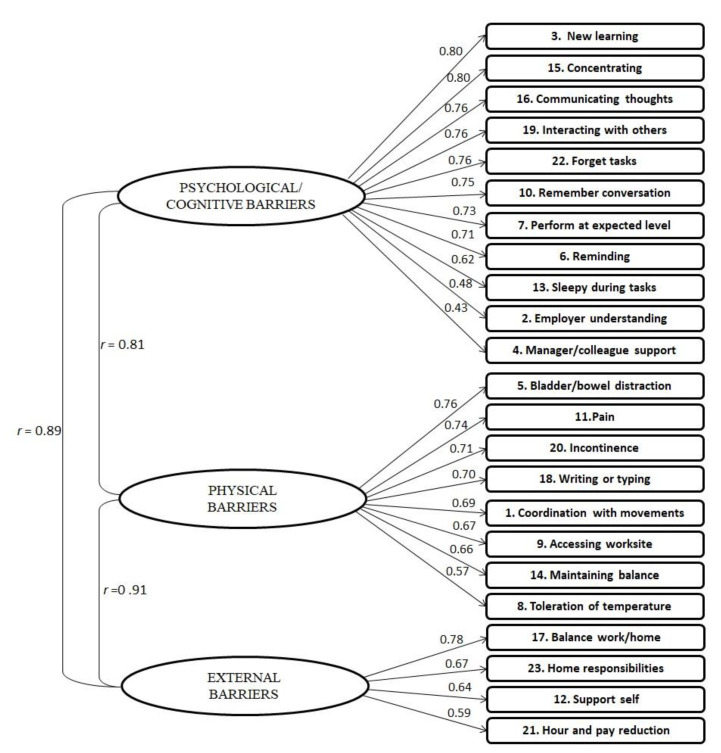
Factor structure of MSWDQ-23. The three latent factors (psychological/cognitive barriers, physical barriers, and external barriers) with their items (depicted in rectangles) and their standardized regression weights (i.e., factor loadings) are shown. The direction of the arrows represents the prediction of observed responses by the latent constructs.

All items had standardized regression weights over 0.3; thus, there was no need for item removal. After inspection of the modification indices, eight error terms within the three subscales were correlated. The final fit statistics of CFA were χ^2^/df = 1.741 (*p* < 0.001), RMSEA = 0.062 (90% lower bound = 0.051, 90% upper bound = 0.072), SRMR = 0.054, and CFI = 0.936, indicating a fair fit of the data (see Figure 1 for factor correlations and item loadings).

### 3.3. Differences of Work Difficulties by Age, Gender, Education, Work Type, and MS Type

Age was positively correlated with psychological/cognitive barriers (rho = 0.199, *p* = 0.005), physical barriers (rho = 0.238, *p* = 0.001), external barriers (rho = 0.247, *p* < 0.001), and total MSWDQ-23 scores (rho = 0.245, *p* = 0.001). The effect of sex on MSWDQ-23 scores was nonsignificant (see Table 2). PwMS with a lower level of education (i.e., primary/secondary) had significantly more physical barriers in their work than those with higher education (i.e., tertiary). Furthermore, PwMS with more work barriers were significantly more likely to work part-time than full-time. Finally, participants with RRMS had significantly smaller MSWDQ-23 scores (i.e., less work barriers) than those with progressive MS (SPMS and PPMS). We considered the above findings as corroborative of the good construct and concurrent validity of the MSWDQ-23.

### 3.4. Floor and Ceiling Effects

Amongst participants, 13.8% had a total MSWDQ-23 score of 0, and none had a score of 100. With regards to the three subscales, 21.4% had a score of 0 for psychological/cognitive barriers, 24.0% for physical barriers, and 33.7% for external barriers, indicating a significant floor effect for all subscales, after using the >15% cut-off. None of the participants had a score of 100 in any subscale. See Table 3 for the MSWDQ-23 descriptive statistics.

### 3.5. Convergent Validity

MSWDQ-23 subscales and total scores were significantly correlated with disease duration, disability, cognitive function, fatigue, MS-related psychological status, and overall health status (see Table 4). Scores showed small-to-moderate correlations with disease duration and EDSS, except physical barriers, which had a large correlation with EDSS scores, as expected. Small-to-moderate correlations were also present between the MSWDQ-23 and cognitive test scores. Moderate-to-large correlations were present between the MSWDQ-23 scores and DASS-21 subscale and EQ-5D scores. On the other hand, the MSWDQ-23 generally had large correlations with the MFIS and MSIS-29 scores.

### 3.6. Internal Consistency

The internal consistency of the overall MSWDQ-23 was excellent (see Table 5 for Cronbach’s alpha values). Internal consistency for the subscales, according to the classification proposed by Kline [38], was excellent for psychological/cognitive barriers, good for physical barriers, and acceptable for external barriers, without any apparent unequal contribution of any one item. The ICC for psychological/cognitive (ICC = 0.991, 95% CI = 0.979–0.996), physical (IC = 0.989, 95% CI = 0.974–0.995), external (ICC = 0.986, 95% CI = 0.967–0.994), and total barriers (ICC = 0.996, 95% CI = 0.990–0.998) was excellent (see Table 5).

## 4. Discussion

By utilizing CFA, this study verified the original three-factor structure of the Greek version of the MSWDQ-23. Furthermore, in accordance with previous validation studies, the Greek version of this instrument showed excellent internal consistency [17,18,19,20,21]. Importantly, there were no missing data, indicating that the Greek version of MSWDQ-23 is a highly feasible instrument. However, there were many participants with zero scores, and thus, there was high risk for a floor effect. It should be noted that the study sample consisted of PwMS currently working and with relatively mild disability (i.e., half of the participants had an EDSS score below 2.0). This rendered the participants less susceptible to work difficulties. Despite this finding, the MSWDQ-23 was significantly associated with other study measures (Table 2 and Table 4), implying the presence of sufficient MSWDQ-23 score variance to produce meaningful associations and, as such, a reduced chance for a floor effect.

Indeed, with increasing age, more work barriers were reported, which is consistent with the expected physical, mental, and cognitive effects of prolonged disease duration, as well as aging [39,40]. Interestingly, the effect of sex was not significant, which was corroborated also by other similar studies [19,21]. This can be ascribed to the gender equality in Greece, such that men and women face similar workplace circumstances. As expected, PwMS with less education faced more physical barriers than those with higher education. Although not tested, we speculated that PwMS who have completed primary/secondary education would be more likely to have jobs requiring physical endurance than those who have completed higher tertiary education, thus explaining the role of physical barriers in PwMS with less education. In addition, this finding might suggest that a lower cognitive reserve reflects less capacity of the brain to adapt neuronally to changing demands due to MS-related disability [41,42]. Part-time workers reported more work barriers than full-time workers. This is consistent with the notion that PwMS, or even their employers, choose to limit their work demands in order to adjust for the high work difficulties [43]. In support of this, previous research has shown that PwMS reporting high work difficulties opt for larger reductions in work hours and may also change their type of work performed [17,20]. Finally, participants with RRMS reported fewer work difficulties than those with progressive MS, most probably signifying the different degree of disability between the two MS groups.

The MSWDQ-23 showed good convergent validity. This was indicated by its significant correlations with disease duration, disability, cognitive function, fatigue, psychological status, and MS-related and overall health status. Higher work difficulties were associated with higher EDSS, poorer cognitive function, more fatigue, stress, anxiety, and depression, and poorer quality of life and health status, a finding that was supported by the results of prior studies [17,18,19,20,21]. Since cognitive changes may predict the long-term clinical evolution of MS [44,45], it was not surprising that they were also associated with working difficulties. Notably, cognitive and psychological measures/barriers were associated with physical barriers/measures and vice versa (Table 4). This is in accordance with previous MS literature showing a strong interplay between work-related quality of life and physical, mental, and psychological health and quality of life in general [2,3,7].

This is the first study using standard cognitive testing (i.e., the BICAMS) and valid patient-reported outcomes (PROs) to explore the psychometric properties of the Greek version of the MSWDQ-23 questionnaire. However, some limitations should be noted. First, we did not use a healthy control group, which would help us ascribe the reported work difficulties to MS. From the available MSWDQ-23 validation studies, only one study used a healthy control group with good discrimination reported [21]; nevertheless, the hypothesis that the reported difficulties extend beyond that experienced by healthy individuals was not tested. Secondly, this study did not examine the future employment status of the participants, which would allow us to confirm the predictive validity of this instrument. However, previous studies have attested to the predictive validity of the instrument for future employment [21,22]. Thirdly, the study did not examine the concurrent validity of the MSWDQ-23 against other similar instruments, due to the absence of similar validated instruments for use in the Greek population. Finally, as mentioned before, half of our participants had mild disability, and this should be taken into account when generalizing the results of this study. Future research should focus on identifying the most important disease-related factors that negatively affect the working capacity of PwMS and on the ability of this instrument to elucidate them. In addition, further studies are needed to explore the predictive validity of the Greek MSWDQ-23 in relation to future changes in vocational status.

## 5. Conclusions

The Greek MSWDQ-23 demonstrated good psychometric properties in PwMS with mild and moderate disability. As such, the instrument can be considered a useful PRO for researchers and health professionals working with PwMS. The instrument also has the capacity to assist professionals working with PwMS and employers to gain valuable insight into the work difficulties faced by PwMS and to facilitate appropriate person-centered or tailored vocational amendments.

## Figures and Tables

**Table 1:** Demographic and disease characteristics of the study population.

***n***	196
Female (%)	126 (64.3)
Mean age (SD, range)	38.6 (10.0, 19–66)
Mean disease duration (SD, range)	7.3 (7.1, 0–36)
Mean education years (SD, range)	15.2 (3.3, 6–27)
Employment	
Full-time (%)	107 (54.6)
Part-time (%)	89 (45.4)
Type of MS	
RRMS (%)	173 (83.3)
SPMS (%)	14 (7.1)
PPMS (%)	9 (4.6)
Median EDSS (SD, range)	2.0 (1.5, 1–7)

MS: Multiple Sclerosis; RRMS: Relapsing–Remitting Multiple Sclerosis; SPMS: Secondary Progressive Multiple Sclerosis; PPMS: Primary Progressive Multiple Sclerosis; EDSS: Expanded Disability Status Scale.

**Table 2:** Differences in MSWDQ-23 scores between patient groups. Values represent means ± standard deviations.

	Male	Female	*p*-Value ^⁋^	Part-Time Work ^¥^	Full-Time Work ^¥^	*p*-Value ^⁋^
Psychological-Cognitive Barriers	16.0 ± 16.6	18.2 ± 18.9	0.528	25.9 ± 20.0	10.0 ± 12.6	<0.001 *
Physical Barriers	17.7 ± 17.0	17.8 ± 21.2	0.360	27.2 ± 22.9	9.6 ± 11.7	<0.001 *
External Barriers	15.8 ± 20.4	21.5 ± 23.2	0.164	30.1 ± 23.8	10.1 ± 15.9	<0.001 *
Total MSWDQ-23	16.6 ± 15.5	18.6 ± 18.8	0.871	27.1 ± 19.4	9.9 ± 11.2	<0.001 *
	**Primary/Secondary Education ^#^**	**Tertiary Education ^#^**	***p*-Value ^⁋^**	**RRMS**	**Progressive MS**	***p*-Value ^⁋^**
Psychological-Cognitive Barriers	20.6 ± 18.9	16.4 ± 17.8	0.172	16.4 ± 17.7	24.8 ± 19.7	0.043 *
Physical Barriers	20.9 ± 18.0	16.8 ± 20.2	0.036 *	14.8 ± 17.9	40.5 ± 18.6	<0.001 *
External Barriers	20.9 ± 23.0	19.0 ± 22.2	0.711	18.0 ± 21.9	30.7 ± 23.0	0.008 *
Total MSWDQ-23	20.7 ± 17.8	17.0 ± 17.6	0.145	16.1 ± 16.9	30.3 ± 18.0	<0.001 *

^#^ Primary/Secondary education was defined as 12 or less years in education. Tertiary education was defined as more than 12 years in education; ^¥^ Part-time job was defined as less than 8 working hours per day. Full time job was defined as 8 or more working hours per day; ^⁋^ Mann-Whitney U tests; RRMS: Relapsing Remitting Multiple Sclerosis; MSWDQ-23: Multiple Sclerosis Work Difficulties Questionnaire-23; * *p* ≤ 0.05.

**Table 3:** Descriptive statistics of the MSWQ-23 subscales and total score.

	Mean	Median	Standard Deviation	Minimum	Maximum
Psychological-Cognitive Barriers	17.4	11.4	18.1	0	70.5
Physical Barriers	17.8	12.5	19.8	0	90.6
External Barriers	19.5	12.5	22.4	0	87.5
Total MSWDQ-23	17.9	12	17.7	0	77.2

**Table 4:** Convergent validity of the MSWDQ-23. Pearson’s rho coefficients (*p*-values).

	Psychological-Cognitive Barriers	Physical Barriers	External Barriers	Total MSWDQ-23
Disease duration (years)	0.243 (0.001)	0.289 (<0.001)	0.198 (0.005) *	0.275 (<0.001)
EDSS	0.361 (<0.001) *	0.614 (<0.001) *	0.395 (<0.001)	0.503 (<0.001) *
SDMT	−0.345 (<0.001) *	−0.357 (<0.001) *	−0.238 (0.001) *	−0.360 (<0.001) *
CVLT-II	−0.245 (0.001) *	−0.244 (0.001) *	−0.141 (0.049) *	−0.247 (0.001) *
BVMT-R	−0.230 (0.001) *	−0.252 (<0.001) *	−0.157 (0.028) *	−0.245 (0.001) *
MFIS	0.778 (<0.001) *	0.752 (<0.001) *	0.712 (<0.001) *	0.831 (<0.001) *
Stress	0.580 (<0.001) *	0.505 (<0.001) *	0.548 (<0.001) *	0.601 (<0.001) *
Anxiety	0.521 (<0.001) *	0.508 (<0.001) *	0.477 (<0.001) *	0.558 (<0.001) *
Depression	0.585 (<0.001) *	0.525 (<0.001) *	0.493 (<0.001) *	0.600 (<0.001) *
MSIS-29	0.717 (<0.001) *	0.822 (<0.001) *	0.688 (<0.001) *	0.823 (<0.001) *
EQ-5D Mobility	0.423 (<0.001) *	0.587 (<0.001) *	0.341 (<0.001) *	0.511 (<0.001) *
EQ-5D Self-Care	0.328 (<0.001) *	0.490 (<0.001) *	0.311 (<0.001) *	0.421 (<0.001) *
EQ-5D Usual Activities	0.471 (<0.001) *	0.608 (<0.001) *	0.474 (<0.001) *	0.573 (<0.001) *
EQ-5D Pain/Discomfort	0.454 (<0.001) *	0.483 (<0.001) *	0.435 (<0.001) *	0.506 (<0.001) *
EQ-5D Anxiety/Depression	0.398 (<0.001) *	0.303 (<0.001) *	0.399 (<0.001) *	0.401 (<0.001) *
EQ-5D VAS	−0.373 (<0.001) *	−0.570 (<0.001) *	−0.452 (<0.001) *	−0.505 (<0.001) *

CVLT-II: California Verbal Learning Test-II; EDSS: Expanded Disability Status Scale; EQ-5D: EuroQuol-5D; MFIS: Modified Fatigue Impact Scale; MSIS-29: Multiple Sclerosis Impact Scale-29; MSWDQ-23: Multiple Sclerosis Work Difficulties Questionnaire 23; SDMT: Symbol Digit Modalities Test; VAS: Visual Analogue Scale; * *p* ≤ 0.05.

**Table 5:** Cronbach’s alpha if item deleted for the MSWDQ-23 subscales.

Psychological-Cognitive Barriers	Cronbach’s Alpha If Item Deleted	Physical Barriers	Cronbach’s Alpha If Item Deleted	External Barriers	Cronbach’s Alpha If Item Deleted
Item 2	0.910	Item 1	0.862	Item 12	0.726
Item 3	0.896	Item 5	0.859	Item 17	0.704
Item 4	0.911	Item 8	0.882	Item 21	0.718
Item 6	0.901	Item 9	0.860	Item 23	0.747
Item 7	0.901	Item 11	0.863		
Item 10	0.899	Item 14	0.858		
Item 13	0.906	Item 18	0.866		
Item 15	0.896	Item 20	0.860		
Item 16	0.899				
Item 19	0.900				
Item 22	0.897				
Total	0.910		0.879		0.778

## Data Availability

The data presented in this study are available on reasonable request from the corresponding author. The data are not publicly available due to privacy reasons.
